# 克唑替尼联合脑转移灶切除、全脑放疗治疗ROS1阳性伴有症状脑转移的肺腺癌

**DOI:** 10.3779/j.issn.1009-3419.2016.08.07

**Published:** 2016-08-20

**Authors:** 敏 张, 立功 聂, 家涌 张

**Affiliations:** 1 100034 北京, 北京大学第一医院放射治疗科 Department of Radiation Therapy, Peking University First Hospital, Beijing 100034, China; 2 100034 北京, 北京大学第一医院, 呼吸和危重症医学科 Department of Pulmonary and Critical Care Medicine, Peking University First Hospital, Beijing 100034, China; 3 100034 北京, 北京大学第一医院, 神经外科 Department of Neurosurgery, Peking University First Hospital, Beijing 100034, China

**Keywords:** 肺肿瘤, 克唑替尼, 脑转移, 手术, 放疗, Lung neoplasms, Crizotinib, Brain metastasis, Surgery, Radiation therapy

## Abstract

**背景与目的:**

伴有脑转移的肺癌预后差。克唑替尼可有效治疗*ROS1*(C-ros oncogene 1 receptor tyrosine kinase)融合基因阳性的肺癌, 但由于血脑屏障通透率较低, 对脑转移灶的治疗效果不佳。本文总结1例综合运用手术、全脑放疗+残留灶补量放疗及克唑替尼等手段治疗*ROS1*融合基因阳性伴有症状脑转移的肺腺癌患者, 并对其有效性及安全性进行讨论和分析。

**方法:**

采用手术切除占位效应明显、引起头疼症状的颅内病灶, 获得病理; 因*ROS1*融合基因阳性, 给予克唑替尼治疗, 250 mg, 2次/d; 术后进行全脑放疗+残留灶补量放疗。按照实体瘤疗效评价标准1.1版(Response Evaluation Criteriation in Solid Tumours, RECIST v1.1)评价客观疗效。按照不良反应通用术语标准4.0版(Common Terminology Criteria for Adverse Events v4.0, CTC AE v4.0)评估用药期间发生的不良事件。

**结果:**

该患者服用克唑替尼3个月后, 肺部病变接近完全缓解(complete remission, CR), 颅内病变部分缓解(partial response, PR), 腹腔病变CR, 视物模糊症状减轻。

**结论:**

综合运用手术、全脑放疗+残留灶补量放疗、克唑替尼治疗*ROS1*融合基因阳性伴有症状脑转移的肺腺癌患者, 可有效控制颅内颅外病灶, 耐受性好。

## Abstract (en)

**Background and objective:**

Lung cancer with brain metastasis had poor prognosis.Crizotinib had been confirmed to be used in *ROS1* (C-ros oncogene 1 receptor tyrosine kinase) rearranged lung adenocarcinoma, but its efficacy in lung cancer with brain metastasis was poor due to the blood brain barrier.In the present study, we reported one case of ROS1 fusion lung adenocarcinoma with symptomatic brain matastasis, who was treated with brain metastases resection, crizotinib, and whole brain radiotherapy plus boost to residual brain metastasis.The safety and efficacy was summarized.

**Methods:**

At first, surgical resection was used to relive mass effect and to biopsy.Then crizotinib (250 mg, *bid*) was chosen for the existence of *ROS1* fusion gene.Whole brain radiotherapy plus boost to residual brain metastasis were used after surgery.Objective response was evaluated by Response Evaluation Criteriation in Solid Tumours (RECIST) v1.1 and brain metastasis were evaluated by computer tomography (CT)/magnetic resonance imaging (MRI) image.Adverse events were evaluated according to Common Terminology Criteria for Adverse Events (CTC AE) v4.0.

**Results:**

After taking crizotinib for 3 months, the lung lesions were close to complete response (CR), the brain metastasis were partial response (PR), the abdomen metastasis were CR and the symptom of blurred vision relieved.

**Conclusion:**

Crizotinib combined with palliative operation and radiation therapy (WBRT plus boost to residual brain metastasis) in the treatment of *ROS1* fusion gene positive lung adenocarcinoma with symptomatic brain metastases, can effectively control intracranial lesions with good tolerance.

肺癌是中国乃至全球发病率和死亡率最高的肿瘤之一^[1]^, 其中85%为非小细胞肺癌(non-small cell lung cancer, NSCLC)。约10%的NSCLC首次就诊时即存在脑转移, 出现脑转移后中位生存时间仅为1个月-2个月。克唑替尼可用于治疗*ROS1*(C-ros oncogene 1 receptor tyrosine kinase)融合基因阳性的肺腺癌^[2-5]^, 但是该药血脑屏障通透率较低, 对脑转移的治疗疗效有限^[6]^。本文报道了1例首诊为*ROS1*融合基因阳性伴有症状的脑转移的晚期肺腺癌患者, 综合运用手术、全脑放疗+残留灶补量放疗、克唑替尼治疗后, 肺部病变接近完全缓解(complete remission, CR), 颅内疗效部分缓解(partial remission, PR), 腹腔病变CR。通过文献复习, 对其安全性和有效性进行分析和讨论。

## 1 临床资料

患者, 女性, 37岁, 不吸烟, 因“头痛、头晕3周, 右侧胸痛、复视1天余”于2015年12月9日就诊于外院, 2015年12月10日头部磁共振成像(magnetic resonance imaging, MRI)检查提示:右侧顶枕交界区、左侧额叶、左额窦旁多发占位, 强化明显, 瘤周水肿明显, 考虑多发转移瘤伴大脑镰下疝征象; 其中病灶最大者位于左额叶, 大小为3.6 cm×2.2 cm×3.1 cm(图 1)。2015年12月18日我院正电子发射型计算机断层显像(positron emission computed tomography, PET)-计算机断层扫描(computed tomography, CT)提示右下肺占位, 高代谢范围31 mm×20 mm×17 mm, 双肺及胸膜下多发小结节, 右侧胸膜结节样增厚, 胰腺高代谢灶, 右锁骨上区、纵隔、右肺门、腹膜后腹主动脉旁多发高代谢淋巴结, 左额叶、右枕叶、左顶枕交界区占位, 考虑右下肺恶性病变伴右侧胸膜转移、胰腺转移、多发淋巴结转移、颅内转移, 伴双肺多发转移可能大。2015年12月22日于我院行颅内占位切除术, 切除左侧额叶病灶, 术后病理提示符合肺腺癌转移, 免疫组化:CK7(+++), TTF1(+++), Napsin A(+++), CK20(+), CDX-2(+), PAX8(-), GATA3(-), GFAP(-)。完善相关分期检查后明确诊断为:右肺下叶中心型腺癌(T4N3M1, Ⅳ期), 多发淋巴结转移(右肺门、纵隔、右侧锁骨上、腹膜后腹主动脉旁), 右侧胸膜转移, 多发脑转移(3个), 胰腺转移, 双肺多发转移可能大。采用突变扩增阻滞系统(amplification refractory mutation system, ARMS)(厦门艾德ADx-ARMS)实时荧光定量PCR法检测*ROS1*融合基因阳性(Exon34阳性, Exon32/35/36阴性, 表 1), 棘皮动物微管相关蛋白样4-间变性淋巴瘤激酶(echinoderm microtubule-associated protein-like 4-anaplastic lymphoma kinase, EML4-ALK)、表皮生长因子受体(epidermal growth factor receptor, *EGFR*)基因、*K-RAS*(Kirsten rat sarcoma viral oncogene homolog)基因的各外显子均未见突变。

**1:**
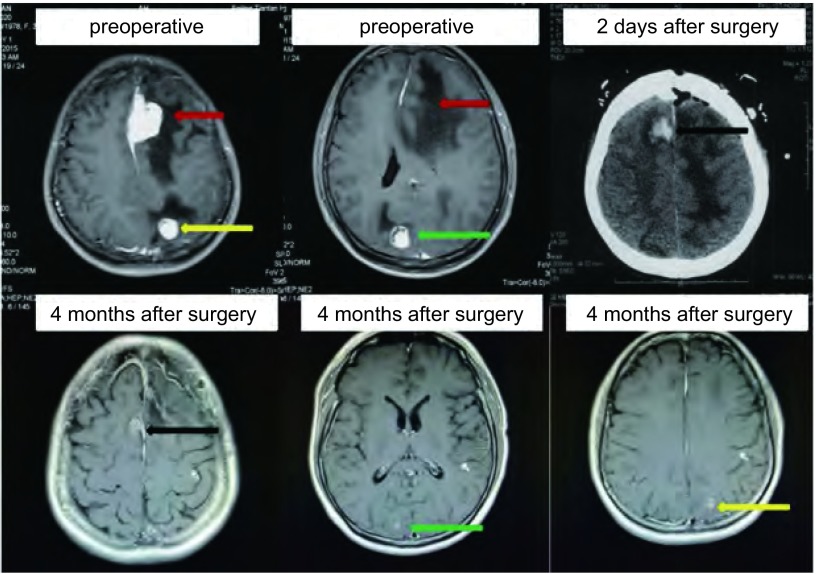
患者治疗前后的头MRI。红箭头:左额叶病灶; 黄箭头:左顶叶转移灶; 绿箭头:右枕叶转移灶; 黑箭头:右额叶出血灶, 术后2天出现。 Magnetic resonance imaging (MRI) of brain before and after treatment.Red arrow:metastatic lesions of left frontal lobe; yellow arrow:metastatic lesions of left parietal lobe; green arrow:metastatic lesions of right occipital lobe; black arrow:hemorrhagic lesion of left parietal lobe occurred 2 days after surgery.

**1:** 实时荧光定量PCR检测ROS1融合基因阳性(Exon34阳性) *ROS1* gene fusion (Exon34) was positive tested by Real time fluorescence quantitative PCR

Type of fusion	Splicing gene/exon	ROS1 splicing exon	Results
*ROS1* variant1	SLC34A2 exon4	Exon32	Negative
*ROS1* variant2	SLC34A2 exon14del		
*ROS1* variant3	CD74 exon6		
*ROS1* variant4	SDC4 exon2		
*ROS1* variant5	SDC4 exon4		
*ROS1* variant6	SLC34A2 exon4	Exon34	Positive
*ROS1* variant7	SLC34A2 exon14del		
*ROS1* variant8	CD74 exon6		
*ROS1* variant9	SDC4 exon4		
*ROS1* variant10	EZR exon10		
*ROS1* variant11	TPM3 exon8	Exon35	Negative
*ROS1* variant12	LRIG3 exon16		
*ROS1* variant13	GOPC exon8		
*ROS1* variant14	GOPC exon4	Exon36	Negative
ROS1:C-ros oncogene 1 receptor tyrosine kinase; PCR:polymerase chain reaction.			

治疗经过:患者于2015年12月22日行颅内占位切除术, 切除左侧额叶病灶, 术后头疼、头晕症状消失, 遗留有视物模糊症状。术后两天复查脑CT提示左额叶占位完全切除, 右额叶邻近术区处可见出血灶(图 1)。因病理检测提示*ROS1*融合基因阳性, 于2016年1月8日开始口服克唑替尼, 250 mg, *bid*。2016年1月12日开始全脑放疗(DT 37.5 Gy/15 f/20 d, 右枕叶、左顶叶转移灶局部加量DT 20 Gy/10 f/14 d), 2016年2月15日放疗结束, 期间持续服用克唑替尼, 2016年1月21日复查脑CT提示颅内转移灶较前缩小, 2016年2月3日复查胸部CT提示胸部病变较前好转。放疗结束后持续服用克唑替尼。3个半月后(2016年4月11日)复查胸CT、头部磁共振成像(magnetic resonance imaging, MRI)、腹部超声, 按照实体瘤疗效评价标准1.1版(Response Evaluation Criteriation in Solid Tumours v1.1, RECIST v1.1)评价客观疗效, 肺部病变接近CR(图 2), 颅内疗效PR(图 1), 腹腔病变CR, 视物模糊症状减轻, 仅夜间存在。服用克唑替尼期间, 主要不良反应为恶心、轻度呕吐(胃内容物), 肝功能异常。呕吐持续约7周后消失。

**2:**
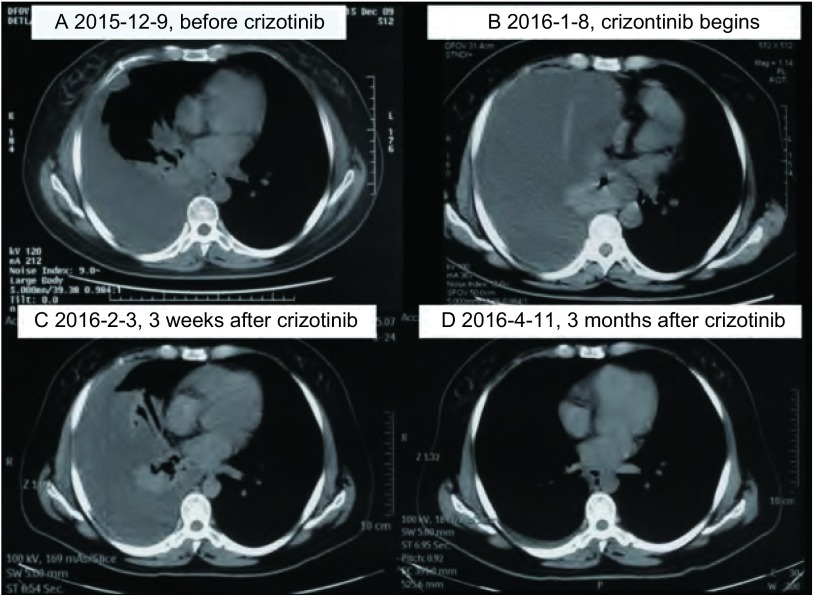
患者临床影像学特征。A、B:患者接受克唑替尼治疗前, 胸部CT显示右肺中心型肿块及大量胸腔积液; C:患者接受克唑替尼治疗3周后, 复查胸部CT显示肿物缩小, 胸腔积液减少; D:患者接受克唑替尼治疗3个月后, 复查胸部CT显示肺部肿块消失, 胸腔积液接近消失, 评效接近CR。 Clinical radiologic features of the patient.A, B:Before crizotinib treatment, chest CT showing mass and miaximal pleural effusion in right lung; C:After 3 wk of crizotinib, CT showing tumors shrinkage and ruduced of pleural effusion and shadow in right lung; D:After 3 month of crizotinib, CT showing disappeared of pleural effusion and shadow in right lung.CT:computed tomography; CR:complete response.

## 2 讨论

肺癌是全球发病率和死亡率最高的肿瘤之一^[1, 7, 8]^, 2011年我国肺癌发病率为48.32/10万, 死亡率为39.27/10万, 发病率和死亡率均居恶性肿瘤的首位^[8]^。NSCLC占肺癌患者的85%左右。约10%的NSCLC首次就诊时即存在脑转移, 在疾病发展过程中脑转移发生率为30%-50%。出现脑转移常意味着治疗效果不佳, 预后极差, 中位生存期约1个月-2个月。

2015年美国国立综合癌症网络(National Comprehensive Cancer Network, NCCN)指南推荐:对有症状的NSCLC脑转移患者, 先治疗脑转移病灶, 然后进行全身治疗。治疗脑转移瘤的手段包括手术、放疗(全脑放疗或者立体定向放射外科)等。既往研究结果显示, 手术治疗可以获得病理标本、迅速有效的缓解颅内占位效应, 还提高生存率^[9-11]^; 手术后全脑放疗较单独放疗的总生存期长(40周 *vs* 15周, *P* < 0.01), 复发率低(20% *vs* 52%, *P* < 0.02)^[12]^, 还有两项随机研究结果^[13, 14]^类似; 对2个-4个脑转移瘤患者, WBRT后肿瘤加量放疗(立体定向放射外科)虽然未能延长生存期, 但可以延长局部复发时间(36个月*vs* 6个月, *P*=0.000, 5)^[15, 16]^。在临床工作中, 一次性手术切除散在颅内的多个病灶, 存在一定的困难, 因而, 手术切除颅内较大占位, 缓解肿瘤占位效应后, 行全脑放疗+肿瘤局部推量放疗是合理的治疗方案。

*ROS1*基因是肺癌的肿瘤驱动基因, 约1%-2.6%的NSCLC出现*ROS1*融合基因阳性^[17, 18]^, 多见于亚裔、不吸烟、女性、腺癌患者^[17-19]^。目前对*ROS1*融合基因阳性是否影响生存尚无定论^[17, 20, 21]^。2015年NCCN指南推荐:对*ROS1*基因重排的肺腺癌患者建议使用克唑替尼进行靶向治疗, 给予克唑替尼250 mg, *bid*^[2-5]^。Ⅰ期临床试验(NCT00585195)的初步结果显示14例*ROS1*阳性的进展期NSCLC患者接受克唑替尼治疗, 第8周时有效率和疾病控制率分别为57%和79%^[22]^。2013美国临床肿瘤学会(American Society of Clinical Oncology, ASCO)年会研究者更新了相关数据, 共33例*ROS1*阳性进展期NSCLC患者入组, 31例接受克唑替尼治疗, 在25例疗效可评价患者中, 总缓解率为56%, 6个月无进展生存率达到71%, 取得了较好的疗效。

但是, Costa等^[6]^报道克唑替尼血脑屏障通透率较低, 对脑转移的治疗效果有限。2015年Lukas等^[23]^报道了1例脑转移患者, 在接受克唑替尼治疗中出现脑转移, 给予低剂量放疗(12.5 Gy/5 f, 因为颅压高症状明显加重而停止放疗)后一直进行克唑替尼治疗, 脑转移灶明显缩小。他们认为, 低剂量放疗可打破血脑屏障, 有助于克唑替尼对脑转移灶的控制。

此例首诊为*ROS1*融合基因阳性伴脑转移的晚期肺腺癌患者, 有明显头疼、头晕症状, 影像学检查提示颅内有3个转移灶, 最大者位于左额叶, 肿瘤最大径达3.6 cm, 瘤周水肿明显, 并有大脑镰下疝征象。考虑到安全性, 避免放疗水肿导致的颅压增高、脑疝等风险, 首先采用手术治疗切除颅内病灶, 迅速有效地缓解占位效应、明确病理诊断。颅内共3个病灶, 散布于左额叶、右枕叶、左顶叶, 不易在一次手术中全部切除, 故仅切除左额叶最大病灶, 术后行全脑放疗+右枕叶、左顶叶小转移灶补量放疗。服用克唑替尼3个月后复查评效:肺部病变接近CR(图 2), 颅内疗效PR(图 1), 腹腔病变CR, 视物模糊症状减轻, 仅夜间存在。

该病例提示我们, 针对*ROS1*融合基因阳性伴有症状的脑转移的肺腺癌患者, 有计划的联合手术、全脑放疗+局部加量放疗控制颅内病变, 破坏血脑屏障, 同时采用克唑替尼控制颅内、颅外病灶, 有可能提高肿瘤治疗效果。这是首次报导联合治疗*ROS1*融合基因阳性伴有症状的脑转移有效的病例。

## References

[1] Guo P, Huang ZL, Yu P (2012). Trends in cancer mortality in China:an update. Ann Oncol.

[2] Shaw AT, Camidge DR, Jeffrey A (2012). Clinical activity of crizotinib in advanced non-small cell lung cancer (NSCLC) harboring *ROS1* gene rearrangement. J Clin Oncol.

[3] Shaw AT, Ou SH, Bang YJ (2014). Crizotinib in *ROS1*-rearranged non-small cell lung cancer. N Engl J Med.

[4] Bos M, Gardizi M, Schildhaus HU (2013). Complete metabolic response in a patient with repeatedly relapsed non-small cell lung cancer harboring *ROS1* gene rearrangement after treatment with crizotinib. Lung Cancer.

[5] Lu S, Azada MC, Ou SH (2015). Choroidal metastasis response to crizotinib in a *ROS1*-rearranged NSCLC patient. Lung Cancer.

[6] Costa DB, Kobayashi S, Pandya SS (2011). CSF concentration of the anaplastic lymphoma kinase inhibitor crizotinib. J Clin Oncol.

[7] Ramalingam SS, Owonikoko TK, Khuri FR (2011). Lung cancer:New biological insights and recent therapeutic advances. CA Cancer J Clin.

[8] Chen W, Zheng R, Zeng H (2015). Annual report on status of cancer in China, 2011. Chin J Cancer Res.

[9] Barker FG 2nd (2004). Craniotomy for the resection of metastatic brain tumors in the U.S., 1988-2000:decreasing motality and the effect of provider caseload. Cancer.

[10] Paek SH, Audu PB, Sperling MR (2005). Reebaluation of surgery for the treatment of brain metastases:review of 208 patient with single or multiple brain metastases treated at on institution with modern neurosurgical techniques. Neurosurgery.

[11] Stark AM, Tscheslog H, Buhl R (2005). Surgical treatment for brain mmetastses:prognostic factors and survival in 177 patients. Neurosurg Rev.

[12] Patchell RA, Tibbs PA, Walsh JW (1990). A randomized trial of surgery in the treatment of single metastases to the brain. N Engl J Med.

[13] Mintz AH, Kestle J, Rathbone MP (1996). A randomized trial to assess the efficacy of surgery in addition to radiotherapy in patients with a single cerebral metastasis. Cancer.

[14] Vecht CJ, Haaxma-Reiche H, Noordijk EM (1993). Treatment of single brain *meta*-stasis:raiotherapy alone or combined with neurosurgery?. Ann Neurol.

[15] Kondziolka D, Patel A, Lunsford LD (1999). Stereotactic radiosurgery plus whole brain radiotherapy versus radiotherapy alone for patients with multiple brain metastases. Int J Radiat Oncol Biol Phys.

[16] Patil CG, Pricola K, Sarmiento JM (2012). Whole brain radiation therapy (WBRT) alone versus WBRT and radiosurgery for the treatment of brain metastases. Cochrane Database Syst Rev.

[17] Bergethon K, Shaw AT, Ou SH (2012). *ROS1* rearrangements define a unique molecular class of lung cancers. J Clin Oncol.

[18] Pan Y, Zhang Y, Li Y (2014). ALK, *ROS1* and *RET* fusions in 1139 lung adenocarcinomas:a comprehensive study of common and fusion pattern-specific clinicopathologic, histologic and cytologic features. Lung Cancer.

[19] Takeuchi K, Soda M, Togashi Y (2012). *RET*, *ROS1* and *ALK* fusions in lung cancer. Nat Med.

[20] Cai W, Li X, Su C (2013). *ROS1* fusions in Chinese patients with non small cell lung cancer. Ann Oncol.

[21] Lee HJ, Seol HS, Kim JY (2013). ROS1 receptor tyrosine kinase, a druggable target, is frequently over expressed in non-small cell lung carcinomas via genetic and epigenetic nechanisms. Ann Surg Oncol.

[22] Shaw AT, Camidge DR, Engelman JA (2012). Clinical activity of crizotinib in advanced non-small cell lung cancer (NSCLC) harboring *ROS1* gene rearrangement. J Clin Oncol.

[23] Lukas RV, Hasan Y, Nicholas MK (2015). *ROS1* rearranged non-small cell lung cancer brain metastases respond to low dose radiotherapy. J Clin Neurosci.

